# The effects of benzimidazole and electrical stimulation on peripheral nerve regeneration after short- and long-term injury

**DOI:** 10.1007/s00418-025-02380-7

**Published:** 2025-04-30

**Authors:** Abubaker El Elhaj, Abdalla Ahmed Eldaw Elamin, Süleyman Kaplan

**Affiliations:** 1 Department of Histology and Embryology, Ondokuz Mayıs University, Samsun, Türkiye; 2 Department of Anatomy, RAK College of Medical Sciences, RAK Medical and Health Sciences University, Ras Al Khaimah, United Arab Emirates; 3 School of Life Science and Bioengineering, Nelson Mandela African Institute of Science and Technology, Arusha, Tanzania

**Keywords:** Benzimidazole, Crush injury, Electrical stimulation, Regeneration, Peripheral nerve, Stereology

## Abstract

This research investigated the effects of benzimidazole (BZ) and electrical stimulation (ES) on peripheral nerve regeneration after short- and long-term injury and assessed functional recovery by means of stereological, histological, and electrophysiological analyses. Fifty-four male albino Wistar rats were divided into nine groups of six animals each. No treatment or surgery was applied to the control (CONT) group. The sciatic nerve was crushed for 5 s in the short-term injury (STI) and for 60 s in the long-term injury (LTI) groups. In the STI + BZ group and the LTI + BZ group, the rats received 25 mg/kg/day of BZ via oral gavage for 28 days. In the STI + ES and LTI + ES groups, a 3-V current was applied for 20 min daily for 28 days. In the STI + BZ + ES group and the LTI + BZ + ES groups, 3-V ES was applied for 20 min per day for 28 days following oral administration of BZ at 25 mg/kg/day for 28 days. All groups were subjected to electrophysiological, electron microscopic, stereological, and statistical analyses. The stereological analyses revealed a significant increases in the numbers of myelinated axons in the STI + ES groups compared with the STI (*p* < 0.01). BZ treatment yielded no significant differences in the numbers of myelinated axons in the groups (*p* > 0.05). Histological evaluation of the STI and LTI groups showed that ES and BZ treatment positively affect the histological structure of the nerve.

## Introduction

Peripheral nerve injury (PNI) refers to partial or complete damage to the peripheral nerve that coordinates activity between the brain, spinal cord, and various other parts of the body (Delibaş et al. 2024; Scholz et al. 2009). PNI impairs motor and sensory functions with symptoms and signs such as numbness, loss of sensation, and paralysis. It may be caused by chronic systemic diseases, traffic accidents, and acts of violence (Scholz et al. 2009). PNI may be caused by direct traumatic injuries and pathophysiological conditions (Robinson 2022). Neurapraxia is a less severe form of damage involving functional loss and absence of Wallerian degeneration (WD). In the event of nerve damage, the axons and the myelin sheath are destroyed with the immediate onset of WD (Faroni et al. 2015). Axonotmesis is reported to be the most common type of treatable PNI. Axonotmesis takes approximately 3 weeks, depending on the damage duration and the nerve’s internal environment. Not all typical nerve structures may be restored after PNI (Alvites et al. 2018), even when medications are used for an extended period. This in turn imposes economic, social, and psychological burdens on patients and their families.

In terms of improved function and muscle reinnervation after PNI, an alteration occurs in the efficiency of nerve impulse conduction activity along the nerve, resulting in an irregular flow of nerve impulses or hyperreflexes. Electric stimulation (ES) therapy enhances physiological nerve activity to promote functional recovery after PNI (Asensio-Pinilla et al. 2009). The degree of regeneration depends on the type and duration of nerve fiber damage, the thickness and disruption of the myelin sheath, and the speed of nerve impulses during muscle contraction. An additional stimulator is needed to accelerate these impulses for them to reach the distal end of the nerve and the target organ (Faroni et al. 2015).

Benzimidazole (BZ) is a chemical compound consisting of heterogeneous benzene and imidazole rings that can help treat spinal cord injury (SCI) (Yu et al. 2019) and neurodegenerative diseases such as Alzheimer’s disease (Faydalı and Arpacı 2024); owing to its potent neuroprotective effects, it could be used in the treatment of peripheral neuropathy and PNI. In this context, the principal idea behind using BZ is to improve peripheral nerve regeneration by restoring the phagocytosis of cellular debris and increasing oxidation at the injury site at the distal nerve end.

A heterocyclic imidazole ring and benzene make up the most promising benzimidazole moieties, which are found in numerous pharmaceuticals with therapeutic use (Salahuddin et al. 2017). Moreover, previous research revealed that benzimidazole possesses antibacterial (Song and Ma 2016), analgesic, and anti-inflammatory activities (Gaba et al. 2014).

ES has electrical and chemical effects on the peripheral nerve to promote repair and build the vascular canal system in the perineurium layer of the peripheral nerve. Increase of blood flow through the nerve affects muscle contraction and relaxation. Thus, ES produces nerve impulses through restoration of the functional components of the PN and the target organ (Gu et al. 2015). ES was tested in the proximal nerve stump of the transacted sciatic nerve of rats to investigate its potential for improving delayed nerve injury repair (DNIR). It showed a significant increase in the number of sensory neurons, the number and diameter of regenerated axons, and the thickness of myelin sheath, indicating that it can promote nerve regeneration in the DNIR by increasing brain-derived neurotrophic factor (BDNF), as a positive contributing factor of the ES on the DNIR (Huang et al. 2013).The application of ES to the distal end of the nerve in diabetic mice has been reported to result in increased blood flow and improved muscle function, thus accelerating muscle contraction (Nakagawa et al. 2017).

PNI causes numerous morphological and physiological changes, depending on its type and duration. Treatment in structural and functional terms should therefore be based on complex substances. The aim of treatment is to increase nerve conduction and impulse transmission and blood flow, resulting in Schwann cell differentiation and macrophage activation (Badri et al. 2017). The present study investigated the effects of BZ and ES on PNI in a rat model. A combination of ES and BZ was studied in terms of good healing and rapid functional recovery of the crushed injured sciatic nerve. Stereological techniques were used to quantify nerve elements, and histological and electrophysiological evaluations were performed to determine the effects of ES and BZ on short- and long-term PNI. This research evaluated the peripheral nerve regeneration in the sciatic nerve of rats following short- and long-term injuries. ES significantly enhanced nerve repair after short-term injury. Notably, light and electron microscopic evaluation revealed positive changes in nerve architecture. The study underscores the importance of electrophysiological techniques, such as ES and electromyography, in nerve injury research. Furthermore, BZ showed limited therapeutic potential for treating crushed nerve injuries. ES is thought to improve peripheral nerve regeneration (PNR) in rat models by stimulating nerve impulses and regulating muscle contraction. BZ, a chemical with neuroprotective properties, is used to assist in nerve fiber biology reconstruction and promote their structural building.

## Materials and methods

### Experimental animals

Fifty-four male Wistar albino rats, aged 13 weeks and weighing 250–300 g, were purchased from the Ondokuz Mayis University (OMU) Animal Experimental Research Center. To focus on sciatic nerve treatment, only male rats were selected to avoid the impact of cyclic hormonal changes on nerve regeneration. The rats were randomly assigned into nine groups of six animals each. Ethical committee of OMU approved this study on date 31 Match 2017 with number 2017/10. All stereological analyses, histological procedures, and electrophysiological tests were performed in the department. The animals were housed in stainless steel and plastic cages at room temperature (21–22 °C) and 45–50% relative humidity. Following PNI, they were given standard commercial chow for 28 days.

### Animal groups

The animal groups in this study were handled as follows: in the control (CONT) group, no surgery or treatment was performed.

In the short-term injury (STI) group, a surgical intervention was performed for exposing the sciatic nerve of the right hind limb between the two parts of the biceps femoris muscle, and a clamp of 50 N was used to crush the nerve on the first day of the experiment for 5 s. In the short-term injury + electrical stimulation (STI + ES) group, after nerve crushing, ES, 3 V was administered for 20 min a day for 4 weeks (3 V/20 min/day/4 weeks) proximally and distally to the site of the nerve injury. In the short-term injury + benzimidazole (STI + BZ) group, after nerve-crushing, each animal received via oral gavage BZ 25 mg/kg/day for 4 weeks. In the short-term injury + benzimidazole + electric stimulation (STI + BZ + ES) group, after nerve crushing, each animal received BZ 25 mg/kg/day for 4 weeks and then ES, 3 V/20 min/day/4 weeks.

In the long-term injury (LTI) group, a surgical intervention was performed, and a clamp of 50 N was used to crush the nerve on the first day of the experiment for 60 s.

**Fig. 1:**
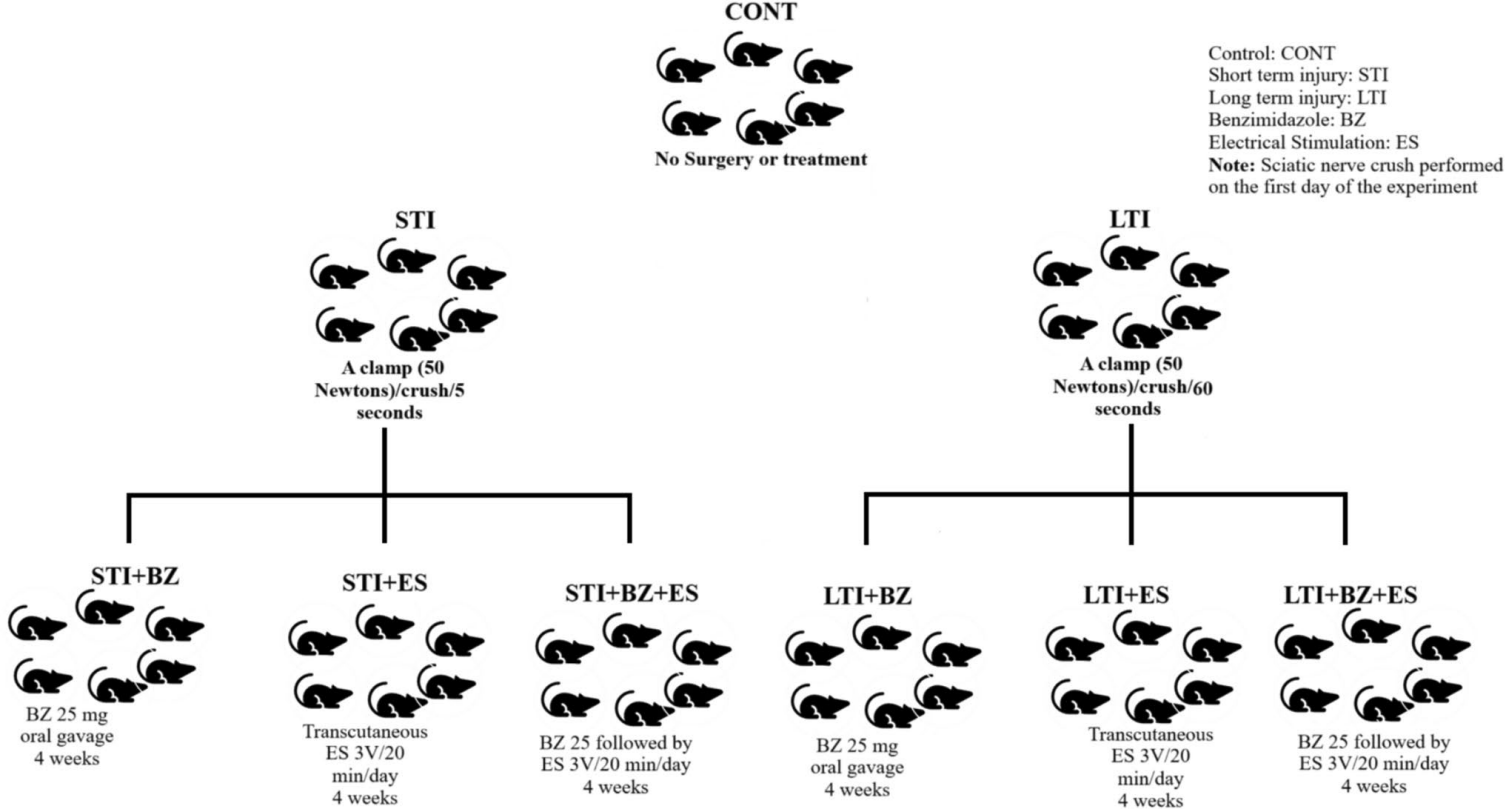
Schematic representation of the experimental groups

In the long-term injury + electrical stimulation (LTI + ES) group, after nerve crushing, ES (3 V/20 min/day/ 4 weeks) was applied proximally and distally to the site of the nerve injury (Penning et al. 2010). In the long-term injury + benzimidazole (LTI + BZ) group, after nerve crushing, each animal received BZ 25 mg/kg/day for 4 weeks. In the long-term injury + benzimidazole + electrical stimulation (LTI + BZ + ES) group, after nerve crushing, each animal received BZ 25 mg/kg/day for 4 weeks and ES, 3 V/20 min/day/ 4 weeks (Fig. 1).

### Surgical techniques

**Fig. 2:**
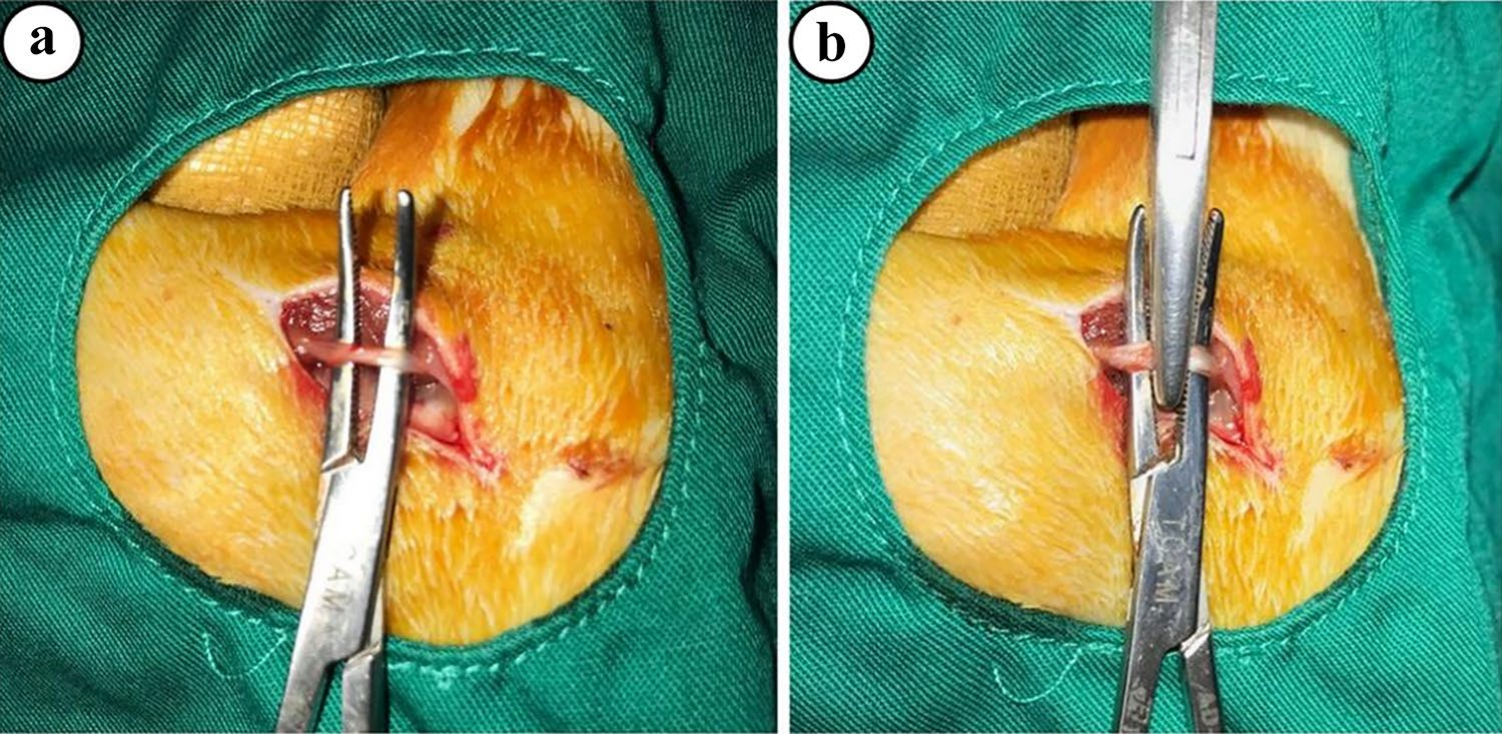
**a** Exposure of the sciatic nerve, **b** Crush injury induced by clamp

All surgical procedures in this study were performed in the Ondokuz Mays University Experimental Research Center. Ketamine (5 mg/kg) (Ketalar^®^, Eczacibasi and Istanbul, Türkiye) and xylazine (2 mg/kg) (Rompun^®^, Bayer and Istanbul, Türkiye) were applied for anesthesia. The animal was first fixed in prone position on the operating table, after which the right hind thigh was shaved (Wang et al. 2014). Povidone-iodine scrub (MEDICA brush; 4% chlorhexidine soap, MEDICA BV, the Netherlands) and povidone-iodine (POVIOD; 10% polyvinylpyrrolidone-iodine complex, Saba, Istanbul, Türkiye) was then applied to the skin of the thigh as a disinfectant. Using a scalpel, a tiny surgical incision was first made on the thigh’s skin.The sciatic nerve was crushed between the two heads of the biceps femoris (Fig. 2).

### Electrical stimulation procedure and sciatic functional index

An appropriately sized plastic container holding one animal was employed for the ES application. The front of the container was opened for ventilation. Two additional openings were used to extract the animal’s right hind legs and tails from the container. An ATABA multi-voltage switching power supply adapter (model AT 13125, Minwa Green Electrics Co. Ltd., Admiralty, Hong Kong) provided the ES. The adapter’s output voltage was adjusted to 3 V. Two electrodes on the adapter stimulated nerve regeneration through the skin (transcuteanous), one placed distal (anode) and the other proximal (cathode) to the damaged area, for 20 min daily for 28 days.

For the application of ES (1-ms pulses, 100 Hz), rectangular silver electrodes measuring 2 × 4 cm were used, and no anesthesia was given during the procedure. A4-size sheets of white paper for sciatic functional index (SFI) and footprint records were stacked on the bottom of a track box following the 28 days of the experiment. The rats were given a path to follow, their paws and hind legs being soaked in Indian blue ink (Brons, İstanbul, Türkiye) to capture their paw prints on the white paper. Numerous prints were collected for each rat, the clearest ones being selected to compute the SFI. A total score of 100 denoted total disability, while a score of zero was regarded as normal if the paw prints were not measurable. It evaluates nerve function by integrating print length, toe spread, and intermediate toe spread (Smit 2006).

### Electromyography

The members of all groups underwent electromyography (EMG) examinations on the last day of the experiment before being sacrificed. The action potential of the nerve impulses passing through the muscle fibers was evaluated using PowerLab4SP (AD Instruments, Sydney, Australia) and EMG Scope software (Version 3.7.2., AD Instruments, Sydney, Australia). The animals were placed under anesthesia before being sacrificed. Ten millimeters away from the sciatic notch, a stimulating hook electrode was affixed, and ground electrodes were attached 2.5 cm from the warning electrode and 1 cm apart on the gastrocnemius muscle surface. Joint action potentials were evaluated using a stimulation voltage of 0.01–10 mV, and uncontrollable movement of the electrode was stopped by grounding. Following surgical nerve dissection, the nerve was stimulated twice, proximally and distally from the graft, using a bipolar steel hook electrode. The tendon-belly technique was used to record the compound muscle action potentials (CMAPs) and for running tests. The nerves were stimulated for electrophysiological examination using a 1 milliampere (mA) current intensity. An electrodiagnostic device was used to measure the distance between the stimulation points and the latency difference to automatically calculate the motor nerve conduction velocity (NCV) between the proximal and distal stimulation sites (mean distance 18 mm).

Axonal loss (AxL) refers to the percentage of axons lost in a damaged neuron compared with the original nerve. It is calculated by dividing the CMAP area difference between operable and normal nerves by the CMAP area of a normal nerve (Van Neerven et al. 2017), as shown in the equation below. The negative peak area under the curve is used to calculate the percentage of axonal loss, which is correlated with elevated AxL levels and is essential for determining the degree of axonal damage in neurological disorders.

$${\mathrm{AxL}}\, = \,\left( {{\mathrm{N}}{-\!\!-}{\mathrm{O}}} \right)\, \div \,{\mathrm{N}}\, \times \,{1}00$$

where AxL is the axonal loss, N is the curve negative peak of the CMAP from the normal nerve, and O is the curve area negative peak of the CMAP from the operated (injured) nerve.

### Preparation of nerve tissue samples for light and electron microscopy

After electrophysiological examinations, when the animals were under anesthesia, 5% glutaraldehyde solution was dropped on the sciatic nerve. Then, the animals were perfused with saline and 10% formaldehyde. After perfusion, part of the sciatic nerve was taken, including the site of the injury and the distal end of the nerve.

The obtained nerves were put into containers of 5% glutaraldehyde solution for 2 weeks. After 4 h, the glutaraldehyde was changed owing to the presence of blood in the fixative solution. During the fixation period, the glutaraldehyde solution was replaced every 3 days. After the fixation period, the nerve segments were washed in the 0.1 M phosphate buffered saline (PBS) (pH 7.4) for 20 min three times. Then nerve segments were washed in the 0.2% glycine to stretch and prevent twisting of the nerves. Thereafter, the nerve segments were returned into PBS for 5 min. Then the nerve specimens were immersed in 2% osmium tetroxide (Sigma, St. Louis, MO) for 2 h in the dark area and were washed again with the same PBS. After the washing process, dehydration was done by using different concentrations of acetone followed by propylene oxide as follows:

15 min in 50% acetone

15 min in 75% acetone

15 min in 95% acetone

20 min in 100% acetone

20 min in 100% acetone

After that, the following stages with propylene oxide were done:

20 min in propylene oxide

20 min in propylene oxide

1 h in 50% propylene oxide + 50% Araldite mixture

Infiltration for 1 h with 100% Araldite

At the end of this process, the tissues were infiltrated in resin (Araldite CY212, Agar Scientific Ltd, Essex, UK) and prepared for embedding. All tissue samples were given code numbers to ensure that the observer performed the stereological analysis in a blinded manner. The sciatic nerve tissues were placed into resin molds at 45 °C. The resin molds were dried overnight, then placed in an oven at 50 °C, adjusted every 30 min to reach a final temperature of 62 °C, and left to dry for 48 h (Kaplan et al. 2023). The tissue blocks were then ready for sectioning using an ultramicrotome (Leica EM UC7, Vienna, Austria). Sectioning was performed using a glass knife. The semi-thin sections (500 nm) obtained were placed onto glass slides and stained with 1% toluidine blue (Sigma-Aldrich Co. LLC., St. Louis, USA), prepared as a mixture of 1% toluidine blue, 2% sodium borate, and 10 ml distilled water for light microscopic examination (Kaplan et al. 2023). The thin sections (70 nm) obtained were stained with uranyl acetate and lead citrate for electron microscopic examination (JEOL JSM-7001F, JEOL Ltd., Tokyo, Japan). Myelinated axons were counted using stereological methods on semi-thin sections (Delibaş and Kaplan 2024).

### Stereological analysis

All nerve tissue samples from the groups were subjected to stereological analysis. The inclusion and exclusion of each parameter during estimation were guided by the unbiased rules of stereological techniques (Delibaş and Kaplan 2024). The two-dimensional fractionator technique was used to estimate the total number of myelinated axons using light microscopic images. The images were taken using an Olympus BX43 light microscope (Olympus Plan C N, 100×/NA = 1.25 oil) with a digital camera (Olympus, Münster, Germany; model SC50, the pixel size: 2.2 × 2.2, 5 megapixels) attached by Olympus U-TV0.5XC-3 (Tokyo, Japan) cellSens Entry software (version 1.14, Oympus, Tokyo, Japan) at various magnifications (Olympus, BX43, Center Valley, PA, USA). To calculate the number of myelinated axons, the two-dimensional fractionator method was applied. The nucleator technique was used for the axonal cross-section area and myelin sheath thickness estimation.

### Statistical analysis

SPSS software was used to analyze the data. The Shapiro–Wilk test was conducted to assess normality. A one-way analysis of variance was used to assess whether statistical significance was found between the groups, and post hoc (Tukey’s honestly significant difference (HSD)) test was then used to determine which groups differed. *p* values less than 0.05 were regarded as indicating significant differences between groups. The data are expressed as mean ± standard deviation (SD).

## Results

**Fig. 3:**
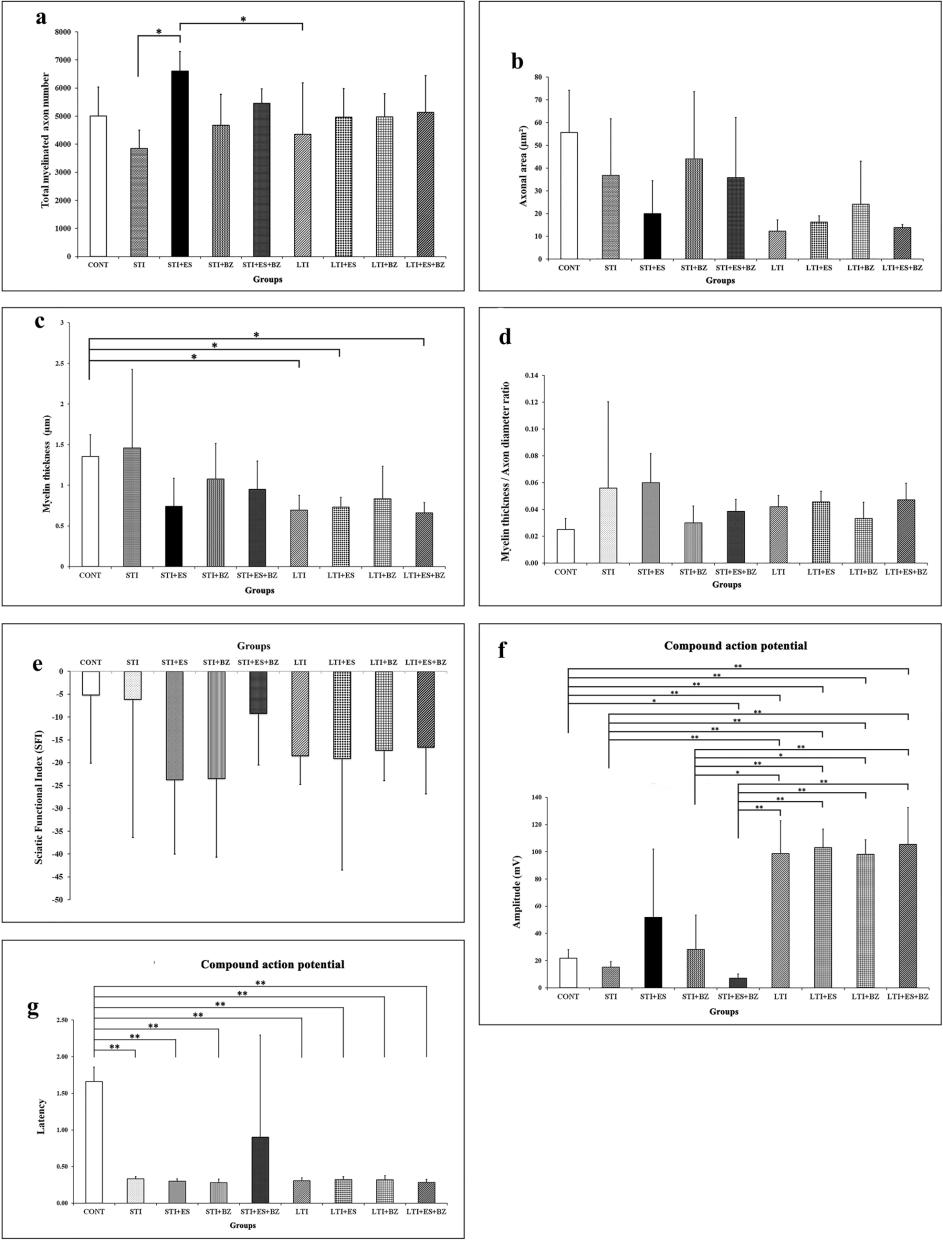
**a** There are notable variations in the sciatic nerve’s mean total number of myelinated axons (*p < 0.05). **b** Mean axonal area comparison between the groups; no significant differences were seen (*p* > 0.05). **c** The mean myelin sheath thickness of nerve fibers in the groups, with a significant difference being observed (*p < 0.05). **d** A comparison of the mean myelin sheath thickness/axon diameter ratios; no significant differences were observed between the groups (*p* > 0.05). **e** There were no significant differences in terms of mean SFI among the groups (*p* > 0.05). **f** Differences in mean compound action potential amplitude values, significant differences at the **p < 0.01 and *p < 0.05 levels. **g** Differences in mean latency; significant differences at the **p < 0.01 level were found between groups

The mean number of myelinated axons was significantly higher in the STI + ES group than in the STI and the LTI groups (*p* < 0.01). However, no significant difference was observed between the STI and the STI + BZ groups or between the LTI and LTI + BZ groups in terms of the mean total numbers of myelinated axons (*p* > 0.05). The LTI + BZ and LTI + ES groups, along with the STI + BZ and STI + ES groups, did not show significant differences. (Fig. 3a).

Evaluation of axon cross-section areas revealed no significant difference between the CONT, STI, and LTI groups (*p* > 0.05). Moreover, no difference was found between the LTI group and the other groups (*p* > 0.05) (Fig. 3b).

The myelin sheath thickness of the myelinated axons exhibited no significant difference between the CONT, STI, STI + BZ, STI + ES, and STI + BZ + ES groups (*p* > 0.05). No significant difference was also determined between the LTI, LTI + BZ, LTI + ES, and LTI + BZ + ES groups (*p* > 0.05). However, a significant decrease in myelin sheath thickness was observed between the CONT and the LTI, LTI + ES, and LTI + BZ + ES groups (*p* < 0.05) (Fig. 3c).

Myelin thickness/axon diameter ratios in the myelinated axons exhibited no differences among any groups (*p* > 0.05) (Fig. 3d).

The stereological analysis of the rat sciatic nerve indicated that the coefficient of error (CE) and the coefficient of variation (CV) for all quantitative assessments demonstrated that the number of animals and the stereological estimates for each group were in an acceptable range except some of them (Table 1). The values of the CV and CE for axon number, axon area, and myelin sheath thickness estimation are given in Table 1.

**Table 1:** Coefficient of errors (CE) and coefficient of variation (CV) values for axon number, axon area, and myelin sheath thickness estimations

	CE and CV values for the mean number of the myelinated axons		CE and CV values for the mean axonal area		CE and CV values for the mean myelin thickness	
Groups	CE	CV	CE	CV	CE	CV
CONT	0.02	0.02	0.02	0.19	0.12	0.18
STI	0.03	0.03	0.02	0.79	0.15	0.77
LTI	0.04	0.38	0.33	0.37	0.08	0.23
STI + BZ	0.02	0.27	0.02	0.67	0.14	0.42
LTI + BZ	0.04	0.15	0.27	0.72	0.05	0.44
STI + ES	0.03	0.35	0.21	0.83	0.07	0.59
LTI + ES	0.04	0.19	0.27	0.15	0.05	0.14
STI + BZ + ES	0.02	0.21	0.03	0.67	0.16	0.33
LTI + BZ + ES	0.04	0.23	0.27	0.08	0.05	0.17

### Electrophysiological findings: sciatic functional index, amplitudes, and latencies

Sciatic functional index (SFI) comparisons between study groups were used to measure the functional recovery of the sciatic nerve. No significant differences in terms of mean SFI values were observed between the STI or LTI group and the control group (*p* > 0.05). No significant functional improvement in terms of mean SFI was observed between the STI group and the STI + BZ, STI + ES, and STI + BZ + ES groups (*p* > 0.05). A comparison of the LTI group with the LTI + BZ, LTI + ES, and LTI + BZ + ES groups showed no functional improvement in the mean SFI (*p* > 0.05) (Fig. 3e).

Statistical analysis of the study data revealed a significantly higher amplitude values in the the LTI, LTI + BZ, LTI + ES, LTI + BZ + ES, and STI + BZ + ES groups compared with CONT group (*p* < 0.01). Additionally, significantly higher amplitude values were found in the LTI, LTI + BZ, LTI + ES, and LTI + BZ + ES groups compared with the STI group (*p* < 0.01). Furthermore, significantly higher values were observed in the LTI, LTI + BZ, LTI + ES, and LTI + BZ + ES groups than in the STI + BZ group (*p* < 0.01). No significant differences were observed between the STI, STI + BZ, and STI + ES groups or between the LTI, LTI + BZ, and LIT + ES groups in terms of amplitude values (*p* > 0.05) (Fig. 3f).

Latency values were significantly higher in the CONT group than in the STI, STI + BZ, and STI + ES groups (*p* < 0.01). Similarly, significantly higher latency values were observed in the CONT group than in the LTI, LTI + BZ, LTI + ES, and LTI + BZ + ES groups (*p* < 0.01). However, no significant variations in latency values were observed between the STI group and the STI + BZ, STI + ES, and STI + BZ + ES groups (*p* > 0.05). The LTI group did not differ significantly from the LTI + BZ, LTI + ES, or LTI + BZ + ES groups (*p* > 0.05) (Fig. 3g).

### Light microscopic findings

**Fig. 4:**
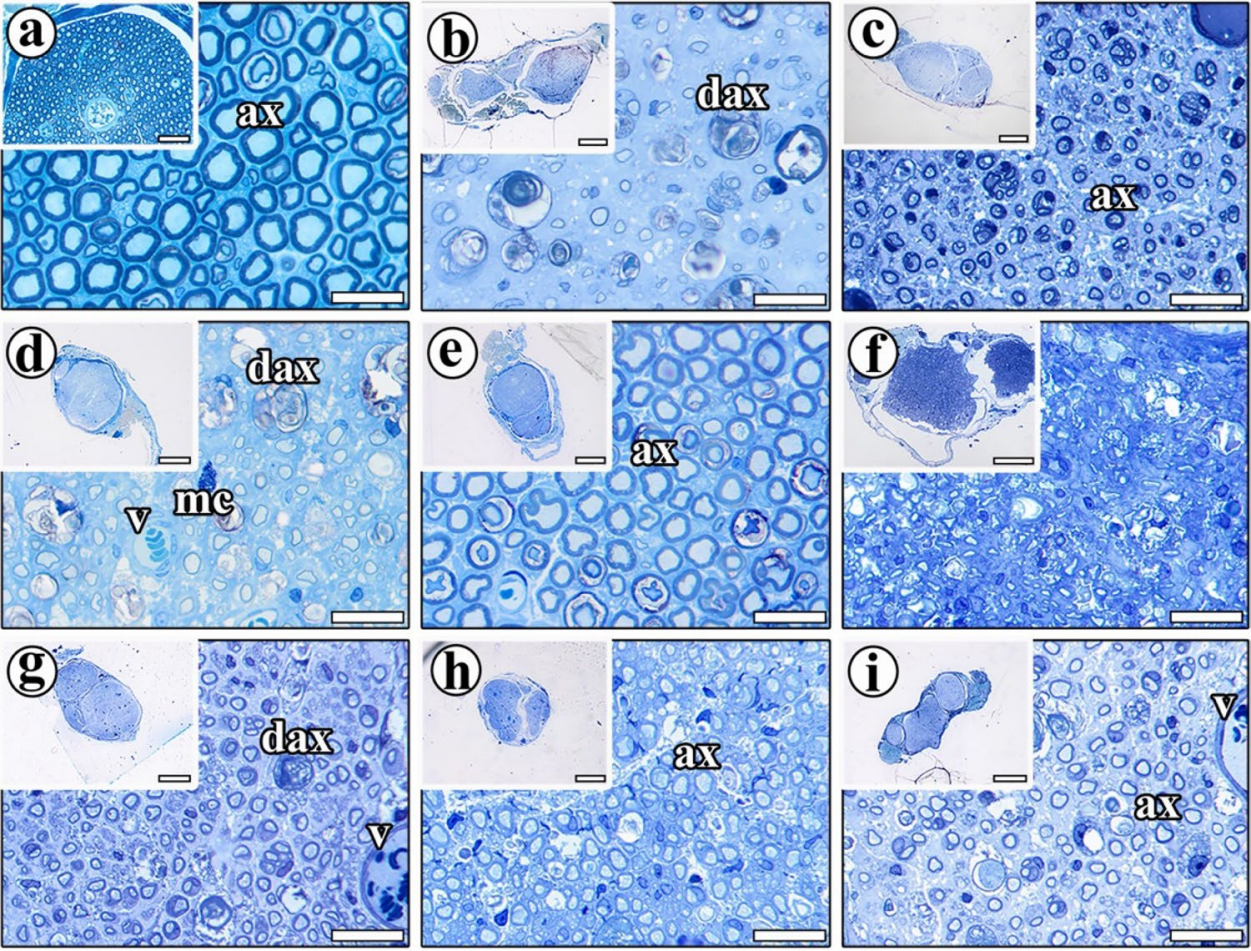
Light microscopic images of the sciatic nerves from the study groups. **a** The myelinated nerve fibers in the **CONT** group exhibit a normal structure (ax, axon). The diameter of nerve fibers is homogeneous, and some nerve fibers have an impaired myelin sheath. **b** Most nerve fiber structures in the STI group were severely impaired (dax, degenerated axon). The image shows a few small-sized axons and large spaces between them that are filled by intercellular tissue substances (ct, connective tissue). **c** Many nerve structures in the STI + ES group were preserved after ES treatment; a thick myelin sheath surrounds the nerve fibers (ax) and healthy structure compared with the injury group. **d** The majority of nerve fibers in the STI + BZ group were not well protected following BZ administration post-injury, although some fibers had a thick myelin sheath and healthy structure. Much axonal debris (dax) and numerous mast cells (Mc) can be seen. The structure of the vessels (v) appears normal. **e** Most nerve fiber structures in the STI + ES + BZ group appear normal after injury and treatment with both ES + BZ. They possess a thick myelin sheath and circular spaces in the myelin sheath. **f** After long-term injury (LTI), most of the nerve structure was filled by connective tissue (ct); it is very difficult to see a cross-section of myelinated nerve fibers. Most of the fibers are very small and difficult to see. **g** Most nerve structures in the LTI + ES group are profoundly impaired after LTI (dax), but some of them have been partly protected after ES. **h** After the injury, most nerves in the LTI + BZ group were filled by connective tissue and intercellular tissue substance. Most newly regenerated fibers have a small diameter after injury treated with BZ. **i** Although most of nerve structures were impaired after injury, many of them were protected by ES + BZ treatment. The formation of a large number of Schwann cells in the injured nerve and a very thin myelin sheath and vessels (v) can be seen in the toluidine blue-stained semi-thin resin section. Scale bars for **a**, **b**, **c**, **d**, **e**, **f**, **g**, **h**, and **i** are 20 µm. Scale bars for the inset of **b**, **c**, **d**, **e**, **g**, **h**, and **i** are 400 µm, for the inset of **f** is 200 µm, and for the inset of **a** is 40 µm

Semi-thin sciatic nerve sections were stained with toluidine blue and examined under a light microscope. The cross-sections of sciatic nerve from the CONT group were first examined. This revealed that the epineurium around the nerve in this group appeared intact, and that the structure of the myelinated nerve fibers was normal. Integrity of axons in nerve fibers and numerous large-sized nerve fibers were seen. Even small-sized nerve fibers can be seen in the nerve cross-section (Fig. 4a).

Examination of cross-sections from the STI group revealed an amorphous substance in most parts of the sciatic nerves. Some parts of the nerve structures were impaired. The axonal regeneration processes depends on the amorphous substances around them, since such an environment creates a medium for the acceleration of nerve sprouts. Very few axons with small diameters were identified, and these were covered by a thin myelin sheath. Macrophages around several axons and myelinated fiber debris were observed after the crush nerve injury. A high quantity of Schwann cells was observed in the areas with newly formed myelinated axons (Fig. 4b).

Cross-sections of the sciatic nerves from the STI + ES group exhibited a normal nerve structure, with a thick, intact connective tissue sheath around them. The distance between nerve fibers is partly decreased. Several spaces filled with amorphous substances were observed between the small-sized axons. The general structures of nerve appeared intact following nerve crush injury and ES treatment. Healthy nerve fibers possessed a thick and regular myelin sheath, and the distance between these nerve fibers was shortened (Fig. 4c).

The sciatic nerve’s overall structure in cross-sections from the STI + BZ group was partly normal in appearance. Numerous small axons were seen, and most of the space between them was filled with amorphous substance. Thick myelin sheaths were found in some nerve fibers, but most were unprotected. The distance between the nerve fibers was significantly increased. Numerous macrophages and mast cells, and many remnants of axons, were observed in these cross-sections (Fig. 4d).

In the STI + ES + BZ group, the combined application of ES + BZ after short-term injury exhibited good protection of the nerve structure. Numerous normal-sized axons were observed, the area between them being filled with a small amount of amorphous substance. The majority of nerve fibers had a thick myelin sheath, and their size was similar to that of the CONT group axons. Although the size of the nerve fibers appeared normal, most myelin sheaths contained a balloon-shaped or circular space. The balloon shape may indicate a low degree of myelin sheath degeneration (Fig. 4e).

In the LTI group, the sciatic nerve morphology was severely impaired. Marked damage was observed in the epineurium. The border of myelinated nerve fibers in the injured nerves was not clearly delineated, possibly of connective tissues that surround them have accumulated. Massive accumulation of Schwann cells surrounded by a foamy cytoplasm of macrophages was also observed (Fig. 4f).

In the LTI + ES group, the nerve retained some of its normal connective tissue sheaths and overall structure. Small-size axons and amorphous substances occupying most of the regions were observed. While some nerve structures were partially protected, most of the morphology was impaired. Numerous Schwann cells were observed in the area, wrapping around the axons and producing the myelin sheath (Fig. 4g).

Cross-sections of the sciatic nerves from the LTI + BZ group revealed that BZ protected the nerve structures after long-term injury, but that the structure of most nerve fibers was destroyed. Myelinated axons were not clearly visible in the injured nerve sections, owing to connective tissue accumulation after injury. The newly regenerated nerve fibers had a small diameter and a thin layer of myelin sheath. Macrophages with a foamy cytoplasm with axonal debris were observed as an empty demyelinated structure. Numerous Schwann cells were observed in the injured nerve (Fig. 4h).

Examination of the sciatic nerve sections from the LTI + ES + BZ group revealed that the general structures were normal. Although the connective tissue sheath surrounding the sciatic nerves was fully protected and some nerve fibers were impaired, most were preserved after BZ + ES treatment. Numerous nuclei of Schwann cells were seen. These surround the axons and produce the myelin sheath to regenerate new nerve fibers (Fig. 4i).

### Electron microscopic findings

**Fig. 5:**
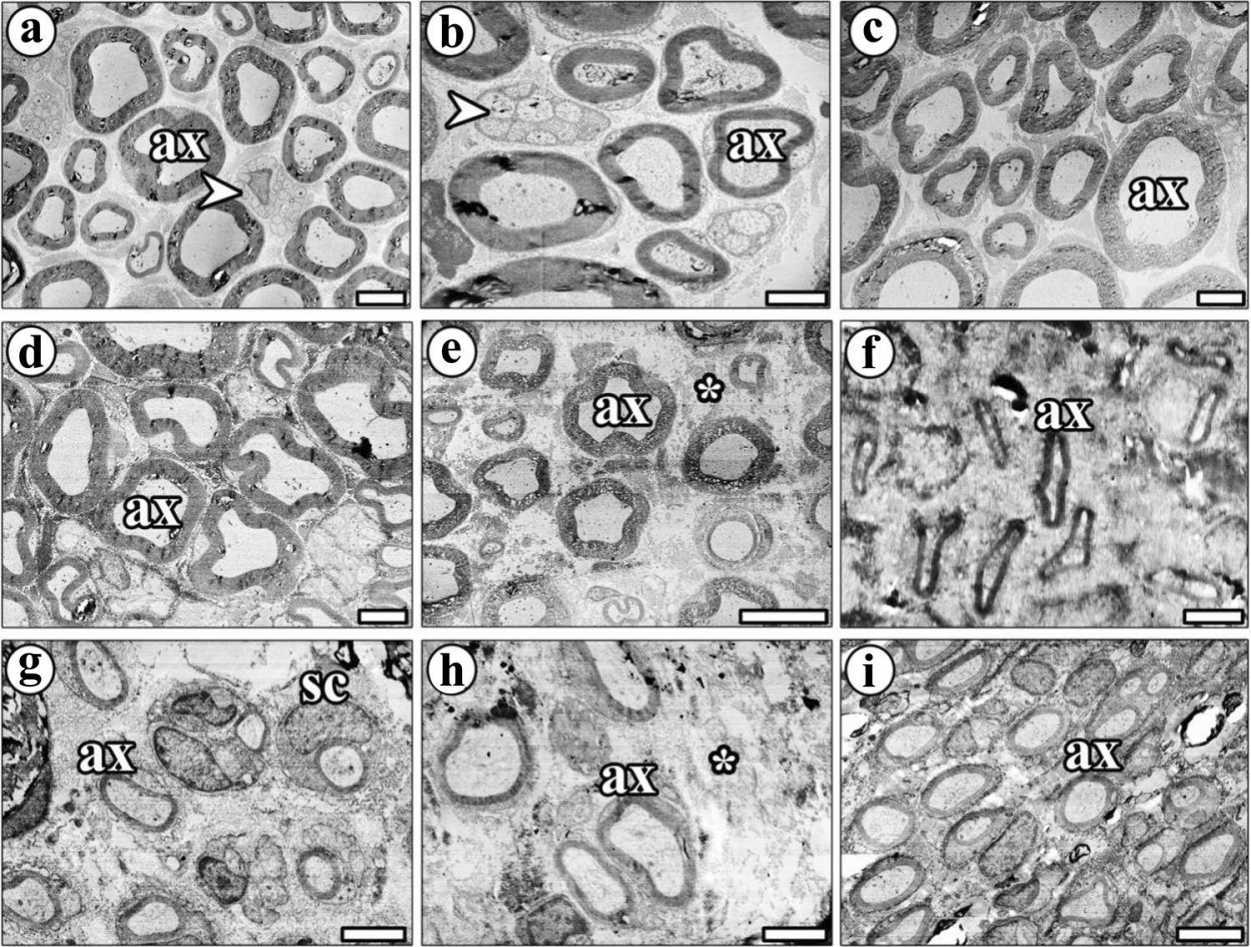
Electron microscopic images of sciatic nerves from the study groups. **a** Images of the sciatic nerves from the CONT group show a well-preserved myelinated (ax) and unmyelinated (arrowhead) nerve fibers. **b** The structure of myelinated (ax) and unmyelinated (arrowhead) nerve fibers appears normal, and both structures are well preserved. **c** Images of sciatic nerves treated with ES after injury shows that the nerve fibers’ structure is unaltered. A thick and well-protected myelin sheath can be seen around the fibers, and the general structure of nerve fibers resembles that of the control group. **d** Following treatment with BZ after injury, the myelin sheath, a thick and well-preserved layer enclosing nerve fiber, appears to be partially normal in structure. **e** The general structures of nerve fibers treated with ES + BZ after the injury are normal in appearance, and a thick, healthy myelin sheath can be seen around them. **f** Connective tissue and cells increased after LTI and few number of nerve fibers with thin myelin sheaths are visible, and nerve fibers lost their integrity. **g** The nerve fibers structure after injury and treatment with ES appears normal. A thick and well-preserved myelin sheath can be seen around small-sized fibers. The borders of each structure in the nerve are clearly visible. **h** The nerve structure appears partly normal following injury and treatment with BZ, although well-preserved thick myelin sheath can be found around them. However, most of the area in the nerve was filled by connective tissue (ct). **i** The general nerve structure looks fine, and healthy thick myelin sheath can be found around them. An increased Schwann cell size, especially in their nuclei, can be seen at the bottom center of the image. Scale bar for **a**, **c** is 4 µm, for **b**, **f** is 2 µm, for **d**, **g**, **h** is 3 µm, and for **e** is 10 µm. (*) connective tissue

Sciatic nerve sections from the CONT group exhibited a normal structure in myelinated nerve fibers, and the nerve vessels were healthy in terms of the endothelium and surrounding tissues. Owing to inadequate fixation, some part of the myelin sheath was lost. Unmyelinated nerve fiber groups were also observed. Inside this collection, the Schwann cell nuclei were visible. Myelinated nerve fibers and surrounding tissues were normal in appearance (Fig. 5a).

Examination of sciatic nerve sections from the STI group revealed an increase in connective tissue and its cells. Loss of nerve fiber integrity and numerous macrophages were observed in this group. Generally, the macrophages possessed a mega-sized cytoplasm rich in myelin debris in the central part of the nerves. Degeneration of myelinated nerve fibers increased after injury (Fig. 5b).

In the STI + ES group, the general nerve fiber structures were normal in appearance following injury and treatment with ES. A thick and well-preserved myelin sheath around the nerve fibers was observed. The unmyelinated axons had distinct borders, and the unmyelinated nerve fibers were intact. Although most myelin sheaths exhibited a normal structure, some small parts were lost. Qualitative evaluation revealed a protective effect of ES on the morphology of injured nerve fibers (Fig. 5c).

In the STI + BZ group, the general nerve fiber structures were normal in appearance following injury and treatment with BZ. A thick and well-preserved myelin sheath was observed around the nerve fibers. In addition, unmyelinated nerve fibers were also intact, the border of each unmyelinated axon being visible. Although most myelin sheaths exhibited a normal structure, some contained balloon-shaped spaces in the sheath (Fig. 5d).

In the STI + ES + BZ group, the general nerve fiber structures exhibited a normal appearance following injury and treatment with a combination of ES + BZ. A well-preserved, thick myelin sheath was observed around the nerve fibers. The unmyelinated nerve fibers were also intact. The connective tissue around the fibers was healthy in appearance (Fig. 5e).

Sciatic nerve sections from the LTI group revealed an increase in connective tissue and its cells post-injury. A significant loss of nerve fiber integrity and numerous macrophages were seen in this group. The characteristic feature of LTI nerves was loss of the fiber borders. The nerves from this group possessed low numbers of nerve fibers with a small diameter and a thin myelin sheath. The shape of the nerve fibers was mostly deformed, and it was also difficult to distinguish the borders of the Schwann cells (Fig. 5f).

In the LTI + ES group, the general structure of the nerve fibers following crush injury and treatment with ES exhibited a normal morphology. However, there was a significant distance between nerve fibers. A healthy myelin sheath was frequently observed around small-sized fibers. It appeared that the ES treatment was effective and provided a better structure compared with the other injury groups. Regenerated nerves contained an increased number of large size Schwann cells. Unmyelinated nerve fibers were also intact (Fig. 5g).

In the sciatic nerve sections from the LTI + BZ group, the structure of nerve tissue was partly normal, with a thick and well-preserved myelin sheath around them. Most sections contained a large amount of accumulated connective tissue. An increased number of Schwann cells was also seen, because of high metabolic activity during the nerve regeneration process. The size of the Schwann cell and their nuclei both increased substantially (Fig. 5h).

In the LTI + ES + BZ group, following injury, the nerves were treated with a combination of ES + BZ, with a normal morphology being observed. A thick and well-preserved myelin sheath was seen around the nerve fibers. Although most myelin sheaths exhibited a normal structure, some very small parts were lost. Numerous remnants of myelinated fibers were seen, resembling black whorls. Large-sized Schwann cells and their nuclei were observed in the sections. Connective tissue was present between these nerve fibers. A combination treatment for nerve injury appears promising compared with the LTI group (Fig. 5i).

**Fig. 6:**
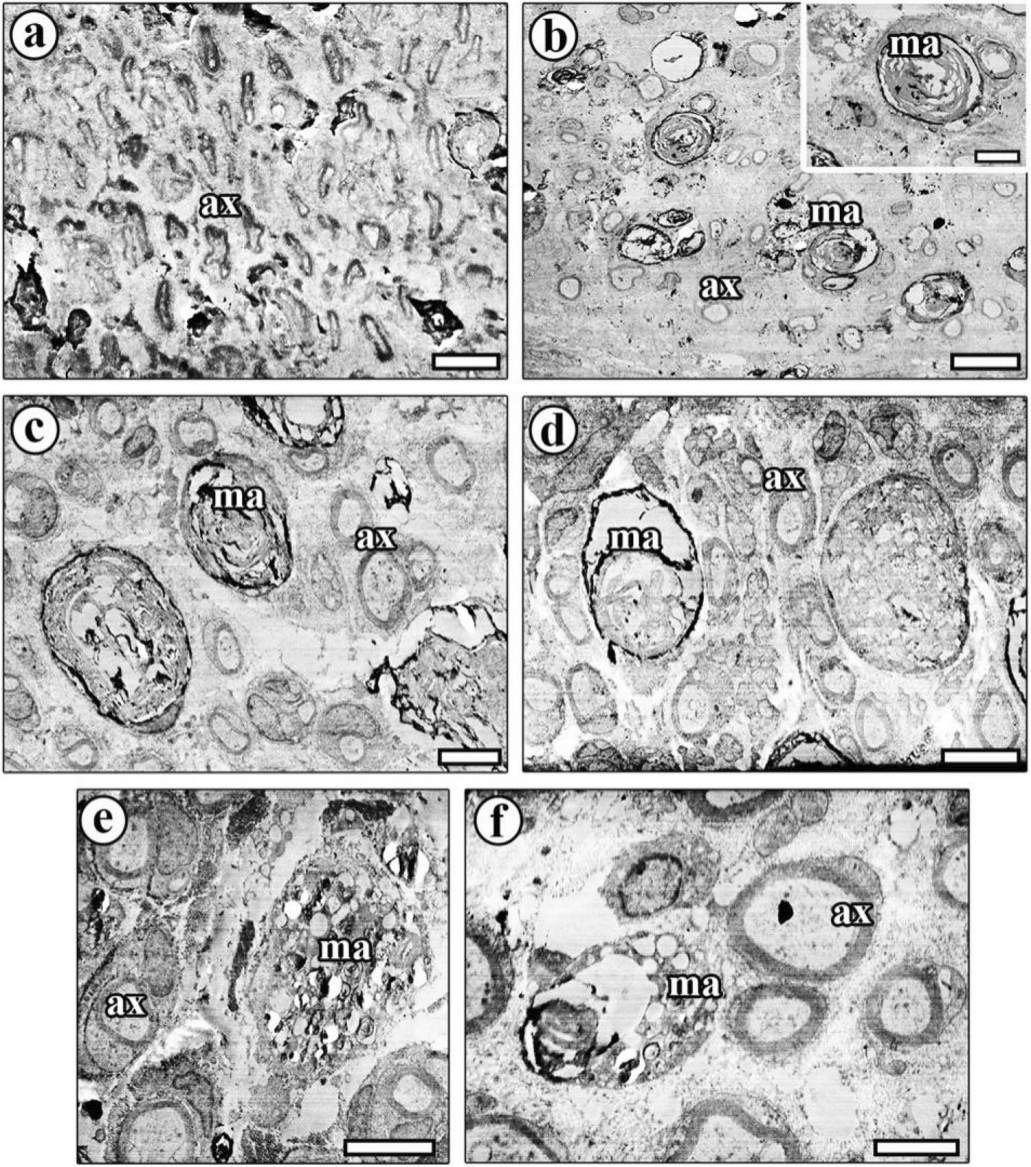
Electron microscopic images taken from the sciatic nerves of all LTI types of groups showing macrophage structures in the injury and after treatment. **a** An image of the only the LTI group is seen; most of the nerve fibers have a considerable distance between each other, and few macrophages are observed. Scale bar 5 µm. **b** After LTI and exposure to BZ, the size and number of macrophages (ma) are increased. A magnified macrophage (ma) is seen in the inset. The scale bar is 10 µm, and for inset is 3 µm. **c**, **d** Applying electric stimulation on the sciatic nerve after LTI creates large-scale macrophages (ma) that engulf debris of nerve fibers in the injured nerve, with sprouting of many new nerves that have intact myelinated nerve fiber structure (ax). The scale bar for **c** is 6 µm, and for **d** is 5 µm. **e**, **f** Exposing the LTI nerve to ES + BZ combination, macrophages (ma) gain large-scale size, and newly formed nerve fibers reach the intact structure of the myelinated nerve fibers (ax). The scale bar for **e** is 5 µm, and for **f** is 3 µm. *Ax* axon, *Ma* macrophage

Increased number and size of macrophages in the LTI sciatic nerve groups after BZ and ES exposure are shown in Fig. 6. On the basis of qualitative electron microscopic image analysis, this was clearly observed. In the LTI group, few macropghages and increased distance between nerve fibers were seen (Fig. 6a), but after ES and BZ types of application on the LTI group, the number of macrophages and their size were seriously increased (Fig. 6b–f).

## Discussion

Damage to the peripheral nerve may impair the nerve impulse transmission through the nerve fibers, leading to weak muscle movement or paralysis in cases of severe injury. The risk of peripheral nerve damage depends on the number of nerve fibers affected and the degree and type of injury. The functional recovery of the nerve is therefore related to the amount and number of damaged nerve fibers, which is largely reflected in the muscle function (Faroni et al. 2015). The return of the muscles to their function and normal activity is usually slow because the restoration of the nerve function is a lengthy process (Eser et al. 2009).

This study investigated sciatic nerve injuries. These were subjected to short- or long-term crush injury. The number of myelinated nerve fibers, the axonal cross-section area, and the myelin sheath thickness of the myelinated fibers were estimated by means of stereological techniques. Following crush injury of the sciatic nerve, the experimental animals received ES, BZ, or both to evaluate their effect on the restoration and regeneration of the sciatic nerve function and structures. Stereological analyses were performed to assess nerve regeneration in terms of morphology, using a two-dimensional fractionator technique to estimate the quantity of myelinated nerve fibres and a nucleator to measure the axonal area and myelin sheath thickness.

ES was applied to the nerve to enhance the repair of the axons and to facilitate the transmission of impulses through the nerve. The results were analyzed, and a positive effect was observed on nerve regeneration. Physiological tests were applied to assess the repaired damage in the sciatic nerve and to evaluate the functional recovery of the muscles by means of the SFI and EMG tests. Functional evaluation of the muscle was done by assessing the duration of nerve impulse transmission in the nerve (latency) within the muscle fibers and the speed of muscle contractility (amplitude). The results of the functional tests were recorded and compared between the different study groups. The results were positive and largely supported the results of the stereological analysis.

The peripheral nerves of the hindlimb and forelimb are extremely commonly employed in crush injury studies. Although the nerve fibers of the peripheral nerve possess considerable potential for regeneration (Delibaş et al. 2024; Mead et al. 2024), the mechanism of recovery is usually unclear, especially in the presence of significant defects in the nerves (Sulaiman and Gordon 2013). Sunderland (1990) reported that pressure on the nerve can lead to neuroma and damage to the epineurium surrounding the nerve fascicle without destroying the nerve fibers in the fascicle.

Numerous changes occur in the nerve in case of injury. These play a role in determining the type of injury and the possibility of regeneration of the affected nerve. Lundborg and Rosén (2007) reported that a number of factors affect nerve restoration, such as the individual’s age and the location and type of injury. Most importantly, the timing of treatment management after injury has a significant impact on nerve recovery. In the present study, we therefore initiated nerve treatment with BZ and ES immediately after nerve crush induction.

Geuna et al. (2009) reported that the integrity of the axons after a nerve injury is critical to the nerve regeneration process. The pressure force used in this study to induce sciatic nerve crush injury was 50 N. This crush injury causes damage to the myelin sheath and axons and also to the connective tissue layers of the nerve (Bridge et al. 1994). The amount of damage to the myelin sheath and the number of affected axons in the crushed nerve depend on the pressure force involved, the duration of pressure on the nerve, and the size of the affected region. Researchers have suggested that the force and duration of the crush on the nerve are directly proportional to the amount of damage to the axons and myelin sheath (Bridge et al. 1994).

Electrical stimulation has been used in rat models of various types of peripheral nerve injury, such as transection and crushing. The immediate use of ES in the treatment of a damaged nerve has been found to promote nerve regeneration (Al-Majed et al. 2000; Ahlborn et al. 2007; Lal et al. 2008; Huang et al. 2009). Significant damage to the peripheral nerve trunk in humans results in slow functional recovery (Belzberg et al. 2004; Palispis and Gupta 2017). In the current study, 3-V ES was applied for 20 min to stimulate regeneration of the crushed nerve. The finding indicates that ES results in effective regeneration after short-term sciatic nerve injury.

Al-Majed et al. (2000) and Brushart et al. (2002) reported that using ES at low frequency for 1 h accelerates nerve regeneration at the suture site. Agnew et al. (1999) also reported that, although ES stimulates nerve regeneration, using it at a frequency greater than 50 Hz could damage the nerve, adversely affecting the remyelination process and axonal growth; ES at a low frequency of 20 Hz for 1 h after surgical repair of a transected digital nerve yielded an improvement in the sensory functions of the nerve (Wong et al. 2015). Slavin (2007) reported that ES was capable of relieving pain in the face and the head resulting from neural causes. Sciatic nerve resection and surgical repair in a rat study revealed that the continuous release of brain-derived neurotrophic factor (BDNF) stimulates the growth of axons and accelerates nerve restoration (Zhang et al. 2000). In the present study, ES exhibited no positive effect in terms of myelin sheath thickness or axonal area, but positively affected axon numbers. Such an effect may be attributed to the release of BDNF to stimulate nerve regeneration (Li et al. 2023).

Nguyen et al. (2007) reported that BZ exhibits therapeutic properties such as anti-inflammatory, antioxidant, immunomodulatory, hormone modulatory, central nervous system stimulating, and lipid control modulating activities. It was therefore used in the present study to assess its ability to regenerate the crushed injured nerve. Treating peripheral nerve damage by antioxidant agents is critical for nerve regeneration. Chen et al. conclude that studies of the effect of BZ on the peripheral nervous system will shed light on its potential effects on nerve regeneration after injury (Chen et al. 2022). Both the BZ and ES interventions share some common benefits, such as promoting nerve regeneration and improving functional recovery. BZ primarily acts by clearing cellular debris, reducing inflammation, and protecting against oxidative stress, while ES works by increasing blood flow, enhancing neuronal survival, and boosting neurotrophic factor levels; together, these effects may offer a holistic approach to peripheral nerve injury treatment.

The critical role of Schwann cells in nerve regeneration and myelin sheath formation is well known. Since BZ has unique features including antioxidant, anticancer, and antibacterial activities, we anticipated that it would exhibit regenerative effects by stimulating the release of neurotrophic factors and increasing Schwann cell activity for remyelination. However, stereological analysis showed no significant differences between the STI and STI + BZ groups or between the LTI and LTI + BZ groups in terms of axon numbers, axonal area, or myelin sheath thickness. We also investigated a combination of BZ + ES treatment to observed its effect on accelerating nerve regeneration. However, no promising results regarding the recovery of the injured nerve were obtained. On the basis of these results, we conclude that BZ is not capable of stimulating nerve recovery.

Electrophysiological tests were conducted to confirm the effects of BZ and ES on peripheral nerve damage. The SFI was adopted as one of the research parameters to assess the quality of peripheral nerve recovery after injury (Sarikcioglu et al. 2009). The SFI test can be performed in one of two ways: conventional ink and paper or digital systems capable of providing accurate results with minimal analytical effort (Lee et al. 2013). The present study applied the conventional SFI method to assess the functional improvement of unilateral sciatic nerve crush injury by collecting the footprints obtained using ink and paper placed on the walking track. After analyzing the data, we compared the SFI values for the crushed right sciatic nerve with those of the normal left sciatic nerve in the same animal. The results showed no statistically significant differences between the two.

A previous study using a rat model to examine the SFI in the context of the protective effect of aminoguanidine in sciatic nerve reperfusion injury reported that the SFI shows a significant improvement over the groups treated with aminoguanidine. Lin et al. (2018) also reported that miconazole promotes nerve regeneration and functional recovery of the crushed sciatic nerve in Sprague–Dawley rats. SFI evaluation revealed a marked improvement in the miconazole-treated groups. However, Ghayour et al. (2017) employed a rat model to investigate the neuroprotective effect of functional restoration of the crushed sciatic nerve. SFI evaluation showed no significant differences between the treated groups. This result is largely consistent with the results of the present study.

The SFI results from this study indicated no positive effects of BZ and ES on peripheral nerve injury. Another functional test applied in this study is EMG, which can be applied to assess the health status of peripheral nerves and voluntary muscles for assessing peripheral neuropathy and muscle disorder. Compound action potential was applied to the gastrocnemius muscles, and peak–peak amplitude and latency values were recorded. Recorded latency values were significantly higher in the CONT group compared with other groups. These results indicate a very slow transmission of nerve signals along the nerve, a sign of impaired nerve function reflected in the muscle movement.

A significant difference in amplitude values was also determined between the CONT group and the STI + BZ + ES group, which exhibited semi-normal muscle contraction due to the presence of regular nerve signals along the nerves in this group. In general, very slow muscle contraction and relaxation were observed in the LTI groups compared with the STI groups, perhaps due to interruptions in the transmission of nerve signals along the nerves in these groups.

Various histological methods, such as histochemical, immunohistochemical, and ultrastructural techniques, can be applied to investigate nerve tissue injuries at the cellular level. Schwann cells, which play a critical role in nerve regeneration, build a new extracellular matrix and form a bridge band of Büngner that directs the growth of the proximal axon segment toward the distal axon segment or in supportive structures such as the epineurium (Geuna et al. 2009; Daly et al. 2012; Bolívar et al. 2020).

Numerous changes in the sciatic nerve have been reported in rats after crush injury. These include decreases in myelinated axon diameters and myelin sheath thicknesses as well as separation of myelin sheath lamellae and axonal cytoplasmic vacuolization (Kaya et al. 2013; Avsar et al. 2014; Helvacioglu et al. 2018). In the present study, the histological structures of the sciatic nerve were examined under a light microscope to demonstrate the success of the regeneration process. In the CONT group, the general structure of the nerve appeared normal. This included the epineurium, myelinated nerve fibers, myelin sheath thickness, and axon diameter. Pronounced impairment of nerve fibers was observed owing to crush injury in the experimental groups compared with the CONT group. Significant morphological alterations in the nerve cross-sections were seen in the STI and LTI groups, although this was observed more evidently in the LTI group. These changes included damage to the epineurium and a decreased myelin sheath thickness and axon diameter. After crush injury, the myelinated nerve fibers could not be observed owing to accumulation of connective tissues and adipose cells in the impaired region. Abundant Schwann cell nuclei enclosed by a foamy cytoplasm of macrophages were observed at the injury site. A previous study reported that one of the reasons for delayed nerve regeneration is nerve vascular interruption after nerve crushing and increased connective tissue (Turgut et al. 2005).

Stereological analysis in terms of quantitative data revealed no positive effects on nerve repair in the LTI + BZ and STI + BZ groups. However, histological examination of nerves revealed a positive effect. Following BZ application, we observed protective effects on the axon diameter and myelin sheath thickness in terms of histopathological evaluation. In the BZ-treated nerves, the epineurium was intact, and numerous myelinated nerve fibers exhibited a preserved structure at morphological evaluation.

In the ES-treated groups, nerve regeneration was better in the STI + ES group than in the LTI + ES group. Myelin sheath thickness and myelinated nerve fiber numbers increased seriously, and ES had a positive effect on nerve regeneration. Moreover, most parts of the epineurium exhibited a normal structure. Very few empty structures representing damaged axons were observed in the regenerated nerve. Finally, on the basis of histological observation, ES appears to stimulate myelin thickness formation and regeneration of the axon in the crush-induced peripheral nerve damage.

Combined treatment in the STI + BZ + ES and LTI + BZ + ES groups exhibited no positive effect based on the stereological analysis in either the short- or long-term injury groups. However, on histological observation, the majority of nerve structures appeared healthy after such treatment. The epineurium around the nerve appeared healthy, and the structure of myelinated nerve fibers was normal in appearance. The diameter of the nerve fibers was also homogeneous. The results of this study showed that ES exhibited more positive effects in case of STI than in LTI, while BZ produced no significant improvement in either STI or LTI on the basis of stereological analysis. Findings of this study contribute to developing effective treatments by exploring the potential of ES and other interventions for enhancing nerve regeneration. These findings could inform clinical approaches for improving recovery in patients with peripheral nerve injuries.

## Conclusions

In contrast to histological examination, the stereological analysis showed that BZ exhibited no regenerative effect on the injured peripheral nerve and has no ameliorative effects in short- or long-term injuries. ES improved the regeneration of the damaged peripheral nerve in short-term injury but not in the long-term injury groups. According to the stereological analysis, using BZ and ES in combination had no positive effect on peripheral nerve regeneration in either short or long-term injuries. However, this was not observed in histological analysis. The SFI and EMG analysis largely supported the histological results. As BZ demonstrated minimal effects on cell proliferation and differentiation, further studies on peripheral nerve regeneration are warranted. The study outcomes highlight that further experimental research on nerve crush injuries offers valuable insights into the use of electrophysiological techniques to enhance nerve regeneration.

## Limitation of the study

The findings of the present study could be supported by immunohistochemical analyses for BDNF and NGF, but we did not have sufficient sources. In addition, high CV values for groups are the most critical shortcoming of this study.

## Data Availability

All datasets used and/or analyzed during the current study are available from the corresponding author on reasonable request.

## Abbreviations

CONT: Control
LTI: Long-term injury
STI: Short-term injury
BZ: Benzimidazole
ES: Electrical stimulation
CV: Coefficient variation
CE: Coefficient of errors
SFI: Sciatic functional index
EMG: Electromyography
NCV: Conduction velocity
AxL: Axonal loss
mA: Milliamperes
CMAPs: Compound muscle action potentials
BDNF: Brain-derived neurotrophic factor
PNI: Peripheral nerve injury
